# Case Report: Kikuchi-Fujimoto Disease: A case of supraclavicular lymphadenopathy

**DOI:** 10.12688/f1000research.19981.2

**Published:** 2021-08-04

**Authors:** Shiza Sarfraz, Hamza Rafique, Hassam Ali, Syed Zawahir Hassan

**Affiliations:** 1 Quaid-e-azam Medical College, Bhawalpur, Pakistan; 2 Internal Medicine, Park Plaza Hospital, Houston, USA

**Keywords:** Lymphadenitis, Cervical lymphadenopathy, Tuberculosis, Lymphoma

## Abstract

Kikuchi-Fujimoto Disease (KFD), also known as histiocytic necrotizing lymphadenitis, is a rare cause of cervical lymphadenopathy. Patients usually present with localized lymphadenopathy, fever and fatigue. Because of the poorly understood etiology, it can be mistaken for an infectious disease or even malignance. Here we discuss a case of KFD that initially presented with left sided cervical lymphadenopathy that later progressed to left supraclavicular lymph nodes. Due to its characteristic overlap with other disorders like tuberculous lymphadenitis and lymphoma, KFD remains an arduous diagnosis for physicians. Therefore, one should be made aware of symptoms that can lead to misdiagnosis in patients.

## Introduction

Kikuchi-Fujimoto Disease (KFD) is known to occur both in the juvenile and adult population. The first case of reported Kikuchi Fujimoto disease was in Japan in 1972 and since then this disease has been described worldwide, with most cases reported in Asia ^1, 2^. Kikuchi-Fujimoto Disease typically follows a benign and self-limited course, characterized by cervical lymphadenopathy (most common). Less frequently, other symptoms might also be present like nausea, weight loss, night sweats and fatigue ^1^. Generally, KFD is diagnosed via excisional lymph node biopsy and histopathological analysis. KFD shares many characteristics with other causes of lymphadenopathy including lymphoma, inflammatory disorders, autoimmune conditions, and infectious causes of lymphadenopathy like tuberculosis infection; therefore, it is important consider KFD in cases of persistent lymphadenopathy and must be differentiated from these conditions ^1, 3^. Treatment is mostly symptomatic with antipyretics, non-steroidal anti-inflammatory drugs (NSAIDS) or on rare occasions, steroids. KFD is associated with spontaneous recovery in 1–4 months ^4, 5^.

## Case presentation

A 25y/o South East Asian male medical student presented in our outpatient department in January 2018 with left-sided cervical lymphadenopathy. The patient reported small bulges along the left side of his neck for one month. Associated symptoms included one month of low-grade fever and fatigue. There was no history of night sweats or reported weight loss. A course of antibiotics two weeks earlier did not improve his symptoms. On presentation, the patient was hemodynamically stable with a temperature of 100.1°C, heart rate of 98 beats/min, respiratory rate was 18 breaths/min and blood pressure was 115/80 mm/hg. On physical examination there was diffuse left cervical and supraclavicular lymphadenopathy. Lymph nodes were rubbery, soft and mobile. There were no changes in hands, eyes or ears. His nose and throat examination were normal. On auscultation of the chest, breath sounds were normal bilaterally and normal heart sounds where present. The abdominal examination was also normal. Initial lab investigations included complete blood count with total and differential leukocyte count, metabolic profile, erythrocyte sedimentation rate (ESR) and lactate dehydrogenase (LDH). This was to rule out any possibility of lymphadenitis, or neoplastic disorder. On laboratory examination there was an increase in lymphocytes percentage (40%) but no leukocytosis and an increase in inflammatory markers including ESR and LDH ( Table 1).

**Table 1.** Laboratory data.

Variable	Reference range	Day 0	Day 30
Hemoglobin (g/dl)	12–16	14.1	13.2
White blood cell count (c/mm)	4000–11000	5000	4600
Red blood cell count (million/c/mm)	3.5–5.5	4.49	4.38
Hematocrit (%)	36–53	42.2	40.9
Mean corpuscular volume (fl)	80–100	94	93.4
Mean corpuscular hemoglobin (pg)	26–34	31.4	30.1
Mean corpuscular hemoglobin concentration (g/dl)	31–37	33.4	32.3
Platelet count (c/mm)	150000–450000	270000	206000
Erythrocyte sedimentation rate (mm/hr)	0–20	60	45
Differential			
Neutrophils (%)	54–62	53	62
Lymphocytes (%)	25–33	40	33
Monocytes (%)	03–07	3	3
Eosinophils (%)	01–06	4	2
lactate dehydrogenase (u/l)	225–400	468	267

A provisional diagnosis of tuberculous lymphadenitis was made based on his occupation. Further investigations were ordered to determine the size and extent of the lymphadenopathy. These included ultrasonography of the neck and abdomen, to visualize any hidden lymphadenopathy that might have been missed during the initial physical examination; chest x-ray, to rule out any active tuberculosis; and interferon-gamma release assay.

**Figure 1.**
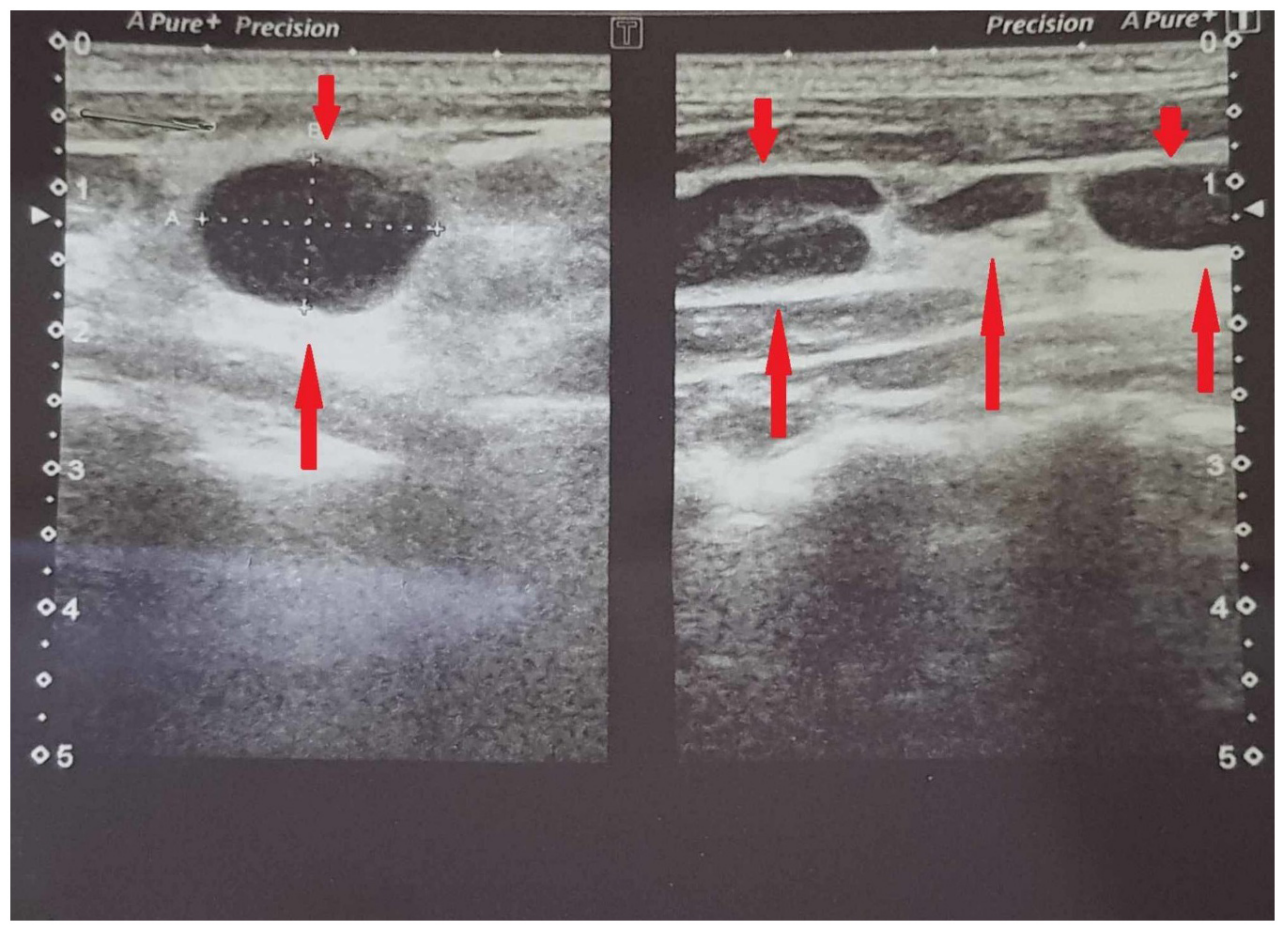
Ultrasound of neck showing enlarged supraclavicular and cervical and lymph nodes (Left side).

On ultrasonography ( Figure 1), the patient showed enlarged multiple discrete left cervical and supraclavicular lymph nodes measuring up to 16×10mm. The rest of the ultrasound report did not show any abnormalities. Chest x-ray was normal and interferon-gamma release assay (IGRA) was not conclusive due to possible delayed transport affecting lymphocyte viability, per the pathology lab. PPD was not not performed as IGRA was done. Anti-tuberculosis therapy was deferred till a formal diagnosis.

To ensure a definitive diagnosis, surgery with lymph node excision and biopsy was performed. An excisional lymph node biopsy from the anterior cervical chain was performed and on histopathological analysis it showed necrotizing lymphadenitis with partial alteration of structure by clusters of histiocytic and interspersed nuclear debris. In preserved areas, lymphoid follicles with pale staining germinal centers were also seen. No evidence of tuberculous granulomas or malignancy was found. Unfortunately, microphotographs could not be procured as testing was done in a third-party laboratory. Stains for acid fast bacteria were also negative. The culture did not show any growth. No further infectious work up was performed.

It was decided that no antibiotics should be given to the patient at this time and watchful waiting was advised. For fever, 500mg paracetamol twice daily was prescribed for one week only. The patient was followed up twice a month in our outpatient clinic to monitor any spread of the lymphadenopathy. The disease course was uneventful. The patient was not given any further medication and watchful waiting was continued. Within two months the lymphadenopathy decreased dramatically, and the patient reported no fever. It completely disappeared in four months.

## Discussion

The actual cause of KFD is still unknown but it has been proposed to have infectious and immunological etiologies ^1^. This disease is thought to be a hyperimmune response to infectious, physical or chemical agents. Some of the unidentified agents may include toxoplasmosis, *Brucella*, *Bartonella henslae*, *Yersinia enterolitica*, human herpes virus, Epstein-Barr virus, parainfluenza, paramyxovirus, parvovirus B19, cytomegalovirus and human immunodeficiency virus ^1, 3– 5^. However, serological and molecular studies have been unable to identify a single specific pathogen. Due to this reason, KFD diagnosis is markedly limited to invasive procedures like excisional biopsy (to observe cellular changes) and not just physical examination and history.

Prevalence of Kikuchi disease has been seen highest amongst the Japanese population and people from East Asia but more recently this disease has been reported all over the world ^2^. Our case is from South Asia, Pakistan.

Typically young adults (aged 20–30) are affected, but it does not seem to spare any age group as cases have been reported in the pediatric population as well, which can be seen in the reports of Byun JH ^6^. However, the case report by Byun JH shows that, when Kikuchi disease occurs in children, it often involves the central nervous system leading to meningitis and encephalitis.

As reported by Deaver *et al.* ^7^, clinical course of this disease has some specific and non-specific features with the specific one being unilateral cervical lymphadenopathy. Although lymphadenopathy is commonly found in cervical lymph nodes other groups such as the axillary and mediastinal lymph nodes may also be involved. Unexplained fever and night sweats are also among the common clinical presentations ^8^. Our patient also presented with all the above listed common complaints. Less common complaints include headache, fatigue, arthralgia, myalgia, night sweats, weight loss, rash and abdominal pain ^8^. Our patient experienced none of them except fatigue. Although rare, patient may present with the involvement of central nervous system and peripheral nervous system ^9^.

Confirmation of diagnosis is done by lymph node biopsy and histopathological analysis which shows distorted nodal architecture. The nodules are mostly necrotic and have debris from nuclear fragmentations due to cellular apoptosis. These necrotic foci are either isolated or clumped together. In addition, there is presence of proliferating histiocytic, T lymphocytes (CD8) and immunoblasts ^7^. The minimum criteria for KFD diagnosis is presence of aggregated histiocytic with occasional crescent-shaped nuclei, plasmacytoid histiocytic, and scattered karyorrhexis ^7^. The biopsy results of our patient were quite similar, making KFD our primary diagnosis. Due to similar clinical characteristics, KFD is often mistaken for lymphoma, tuberculosis, systemic lupus erythematosus and even metastatic adenocarcinoma. Therefore, any physician who comes across a case of lymphadenopathy, should keep KFD in mind when consider differential diagnoses. KFD is self-limiting and resolution occurs is one to four months. There are no specific drugs for KFD and usual treatment is symptomatic, consisting of antipyretics and analgesics.

## Conclusions

We describe a case of Kikuchi-Fujimoto Disease, a self-limiting necrotizing lymphadenitis that started with cervical lymph node swelling but progressed to left supraclavicular lymph node involvement, which makes it unique. Recognition of this disease is important as it can mimic lymphoma or even metastatic adenocarcinoma. Early diagnosis and treatment can help avoid unnecessary testing and improper treatments among patients. Correctly recognizing the symptoms of KFD can also save one from the emotional stress of misdiagnosis.

## Consent

Written informed consent for publication of their clinical details and clinical images was obtained from the patient.

## Data availability

All data underlying the results are available as part of the article and no additional source data are required.
