# Supplementary material for: Case Report: Kikuchi-Fujimoto Disease: A case of supraclavicular lymphadenopathy
Source: F1000Res. 2021 Aug 4;8:1652. Originally published 2019 Sep 17. [Version 2] doi: 10.12688/f1000research.19981.2 (PMC8343398; doi:10.12688/f1000research.19981.2)
Supplement: Peer review history

# Peer review: Reviewer response for version 2

**DOI:** 10.5256/f1000research.58501.r91184

**Published:** 2021-08-05

**Authors:** Karl O. A. Yu

**Affiliations:** 1 Division of Infectious Diseases, Department of Pediatrics, University at Buffalo, Buffalo, NY, USA

The workup in this case is not as exhaustive as I would have preferred (e.g., a PPD was not done, and an IGRA was not repeated after being inconclusive), but I doubt the diagnosis or disease management would be changed.  

 A good addition to the clinical literature on presentations of necrotizing lymphadenitis / Kikuchi-Fujimoto disease.

Are enough details provided of any physical examination and diagnostic tests, treatment given and outcomes?

Partly

Is the case presented with sufficient detail to be useful for other practitioners?

Yes

Is sufficient discussion included of the importance of the findings and their relevance to future understanding of disease processes, diagnosis or treatment?

Yes

Is the background of the case’s history and progression described in sufficient detail?

Yes

Reviewer Expertise:

infectious disease; immunology; pediatrics; host/pathogen interactions

I confirm that I have read this submission and believe that I have an appropriate level of expertise to confirm that it is of an acceptable scientific standard.

# Peer review: Reviewer response for version 1

**DOI:** 10.5256/f1000research.21930.r62892

**Published:** 2020-05-12

**Authors:** Syed Hamza Bin Waqar

**Affiliations:** 1 Internal Medicine, Civil Hospital Karachi, Dow University of Health Sciences, Karachi, Pakistan

- Needs to improve the verbal/grammatical check.. as malignancy, not malignance.
- Run a grammar check thoroughly; check for repetition.
- To rule out neoplastic disorder, you need to get an excisional biopsy - not perform blood tests (case presentation p1).
- Add any serological tests like toxo, hiv, csd, cmv-ebv etc. that you might have done to rule out infectious causes of lymphadenopathy apart from IGRA which the authors have mentioned already.
- Add any rheumatological tests like ANA etc. that might have been done to rule it out before proceeding to excisional biopsy.
- Any rash that the pt might have/haven't noticed should be mentioned.
- How does the involvement of supraclav lad make the case unique?

Final Comments:

 Authors have done a great job in summarizing KFD and have beautifully presented the case. With only a little alteration in the case presentation by adding serological markers will make the case look more complete - although it's not necessary.

Are enough details provided of any physical examination and diagnostic tests, treatment given and outcomes?

Partly

Is the case presented with sufficient detail to be useful for other practitioners?

Yes

Is sufficient discussion included of the importance of the findings and their relevance to future understanding of disease processes, diagnosis or treatment?

Yes

Is the background of the case’s history and progression described in sufficient detail?

Yes

Reviewer Expertise:

Internal Medicine.

I confirm that I have read this submission and believe that I have an appropriate level of expertise to confirm that it is of an acceptable scientific standard.

# Peer review: Reviewer response for version 1

**DOI:** 10.5256/f1000research.21930.r56268

**Published:** 2019-11-07

**Authors:** Karl O. A. Yu

**Affiliations:** 1 Division of Infectious Diseases, Department of Pediatrics, University at Buffalo, Buffalo, NY, USA

Sarfaz *et al. *report a rare (but probably it is not-so-rare) case of Kikuchi-Fujimoto lymphadenitis in a young adult.  (disclosure:  I just co-authored a recent case series on KFD).   I am glad for this report to go out, as I suspect, based on my anecdotal experience, that this is not as rare as people think it is,  but is probably under-diagnosed/under-reported.  Seeing more reports as this in the literature may change conventional opinion.

 I have a few suggestions to improve the manuscript:

- The authors mention that testing for tuberculosis was done.  I would suggest clarification as to why the IGRA was inconclusive.  Was there a high background, or a failed mitogen testing.  If the former -- this may be evidence for baseline inflammation (principally, IFN-gamma/Th1 inflammation) in the patient's blood at that time -- this is potentially important.  If the latter, this may be due to shipping issues, or evidence for transient anergy or other immunosuppression.
- The authors say stains for acid fast bacilli were done and were negative.  Was there a culture?
- Was a PPD checked?
- The authors state that histopathological confirmation of the diagnosis was made.  Are microphotographs available for the reader themselves?
- The authors state that the patient has "high lymphocytes."  The patient's absolute lymphocyte count (2000 / uL) is NOT high.
- "Epstein-Barr" is misspelled.
- While not necessarily needed, since the authors did talk about the different infections associated with KFD, it would be enlightening to see what infectious workup was done for this patient, apart from an IGRA and an AFB stain.
- With the suspicion of tuberculous meningitis, please clarify if anti-tuberculous therapy was started.
- The authors also state that the case from from "South East Asia, Pakistan."  I know that many Pakistanis and Indians are taught that their country is in southeast Asia -- but that is not the usual convention outside of Pakistan or India.  Southeast Asia is conventionally the 10 states of ASEAN plus East Timor and Papua New Guinea.  I would suggest the authors use "south Asia", or just say "Pakistan" to avoid any ambiguity.

Are enough details provided of any physical examination and diagnostic tests, treatment given and outcomes?

Partly

Is the case presented with sufficient detail to be useful for other practitioners?

Yes

Is sufficient discussion included of the importance of the findings and their relevance to future understanding of disease processes, diagnosis or treatment?

Yes

Is the background of the case’s history and progression described in sufficient detail?

Yes

Reviewer Expertise:

infectious disease; immunology; pediatrics; host/pathogen interactions

I confirm that I have read this submission and believe that I have an appropriate level of expertise to confirm that it is of an acceptable scientific standard, however I have significant reservations, as outlined above.
